# Source-Specific Oxidative Potential of PM_2.5_ in Xi’an: Roles of Water-Soluble Metals Revealed by DTT Assay and Interpretable Machine Learning

**DOI:** 10.3390/toxics14080646

**Published:** 2026-07-23

**Authors:** Lei Chen, Na Wang, Qian Zhang, Xinghua Zhang, Zhihua Li, Weidong Jing

**Affiliations:** 1 School of Environmental and Municipal Engineering, Xi’an University of Architecture and Technology, Xi’an 710055, China; 13891983388@163.com (L.C.); rb_fcosuiqyh@163.com (N.W.); zhangqian2018@xauat.edu.cn (Q.Z.); lizhihua@xauat.edu.cn (Z.L.); 2 Department of Ecology and Environment of Shaanxi Province, Xi’an 710006, China; 3 Shaanxi Key Laboratory of Earth Surface System and Environmental Carrying Capacity, College of Urban and Environmental Sciences, Northwest University, Xi’an 710127, China; zhangxinghua@nwu.edu.cn

**Keywords:** PM_2.5_, oxidative potential, water-soluble metals, DTT assay, source-specific toxicity, machine learning

## Abstract

Oxidative stress is a central mechanism underlying the toxicity of fine particulate matter (PM_2.5_); however, the source-specific chemical drivers of particle-associated oxidative potential remain incompletely understood. In this study, the oxidative potential (OP) of ambient PM_2.5_ in Xi’an was investigated during winter and summer using the dithiothreitol (DTT) assay, with particular emphasis on the toxicological roles of water-soluble metals and emission sources. PM_2.5_ exhibited significantly higher volume-normalized OP (DTT_v_) in winter, indicating an enhanced particle-associated oxidative stress burden during the heating period. Notably, although water-soluble metals accounted for only a minor fraction of PM_2.5_ mass, interpretable machine learning analysis (XGBoost–SHAP) identified potassium and manganese as dominant contributors to OP, highlighting the importance of biomass burning tracers and redox-active transition metals in particle-mediated reactive oxygen species generation. Source apportionment further revealed pronounced seasonal contrasts: dust sources contributed substantially to wintertime OP primarily due to their large mass loading, whereas traffic-related emissions dominated OP in summer owing to their high intrinsic oxidative toxicity. Overall, these findings suggest that variations in PM_2.5_ oxidative potential are more closely associated with chemical composition and source-specific oxidative activity than with particle mass alone, providing additional insight into the factors influencing PM-related health risks.

## 1. Introduction

Particulate matter (PM) with an aerodynamic diameter ≤ 2.5 μm (PM_2.5_) represents a major environmental risk factor for human health [1]. Due to its small size, PM_2.5_ can penetrate deeply into the respiratory tract and deposit in the alveolar region, where it induces a range of adverse biological effects, including inflammation, epithelial barrier dysfunction, and epigenetic alterations. Consequently, these toxic effects have been linked to the development and exacerbation of various pulmonary diseases, such as chronic inflammatory lung diseases, cancer, and respiratory infections [2,3,4].

The toxicity of PM_2.5_ varies substantially depending on its sources, chemical composition, and atmospheric aging processes. Therefore, relying solely on PM_2.5_ mass concentration is insufficient to characterize its health impacts [5,6,7,8]. Among the multiple toxicological pathways triggered by PM_2.5_ exposure, oxidative stress is widely recognized as a central mechanism underlying PM-induced cytotoxicity [9]. In particular, excessive generation of reactive oxygen species (ROS) can disrupt cellular redox homeostasis, leading to oxidative damage of lipids, proteins, and DNA, as well as the activation of inflammatory and cell death pathways [10,11,12]. The ability of PM to generate ROS and deplete antioxidants is defined as oxidative potential (OP).

Oxidative potential (OP), defined as the capacity of PM to generate ROS or deplete antioxidants, has been proposed as a toxicologically relevant indicator linking chemical composition of PM_2.5_ to oxidative stress–mediated health effects [13,14,15]. Compared with PM mass concentration or individual chemical components, OP provides a more integrative measure of particle toxicity and has received increasing attention in recent years [16,17,18]. OP can be quantified using both cell-based and acellular assays. Although cell-based methods offer biological relevance, acellular assays are more suitable for large-scale studies due to their lower cost, higher reproducibility, and ease of standardization [19].

Among acellular methods, the dithiothreitol (DTT) assay is one of the most widely used approaches for measuring OP, as it is sensitive to a broad range of redox-active PM components [19,20]. Specifically, the DTT assay quantifies the rate at which PM consumes the reducing agent DTT, reflecting the particle’s capacity to induce oxidative stress. OP values are commonly expressed as mass-normalized DTT activity (DTT_m_), representing intrinsic particle toxicity, and volume-normalized DTT activity (DTT_v_), reflecting population-relevant exposure risk [21,22,23]. However, although OP is considered to be a better health indicator, several scientific gaps remain in the current research, particularly regarding regional differences, temporal variations, and changes in chemical composition and source distribution, all of which suggest that PM_2.5_ toxicity is not solely determined by particle mass concentration [24,25,26].

Previous studies have identified several PM components associated with OP, particularly water-soluble transition metals and combustion-related species. For example, metals such as Fe, Cu, Mn, and Zn can catalyze Fenton and Fenton-like reactions, leading to ROS generation, while K is commonly associated with biomass burning emissions that contain redox-active organic compounds [10,11,12,13,14]. However, the relationships between OP and individual chemical components are often nonlinear and influenced by complex interactions among species, which are difficult to resolve using conventional linear statistical approaches [27,28]. In addition to chemical composition, emission sources play a critical role in determining PM_2.5_ oxidative toxicity. Different sources may contribute similarly to PM_2.5_ mass but differ substantially in their ability to induce oxidative stress.

Traditional PMF-based source apportionment approaches are effective for quantifying source contributions to PM_2.5_ mass and oxidative potential. However, they provide limited information regarding the specific chemical constituents responsible for source-dependent toxicity. Moreover, PMF–MLR frameworks generally assume linear relationships between source contributions and OP, potentially overlooking nonlinear effects and interactions among redox-active species. Consequently, the mechanistic links between PM composition, emission sources, and oxidative potential remain incompletely understood [29,30,31,32].

Despite increasing evidence linking PM_2.5_ oxidative potential to chemical composition and emission sources, the specific roles of water-soluble metals and source-related toxicity remain insufficiently understood, particularly in heavily polluted inland cities of northwestern China. Moreover, conventional statistical approaches often fail to capture the nonlinear relationships between oxidative potential and PM constituents. Therefore, this study combined DTT-based oxidative potential measurements, XGBoost–SHAP analysis, and PMF source apportionment to identify the dominant water-soluble metal drivers and source-specific contributions to PM_2.5_ oxidative potential in Xi’an. The findings provide mechanistic insights into the determinants of PM-induced oxidative stress and support source-oriented air pollution control strategies.

## 2. Materials and Methods

### 2.1. Sample Collection

PM_2.5_ filter samples were collected from the rooftop of the School of Environment, Xi’an University of Architecture and Technology, Shaanxi Province. No significant buildings or emission sources were present within 1 km of the sampling site. The surrounding 5 km area consists mainly of educational, administrative, and residential zones, characterized as a densely populated commercial-residential district without any notable local emission sources. PM_2.5_ samples were collected during winter (December 2021 and December 2022; *n* = 46) and summer (July 2021 and July 2022; *n* = 37). Sampling was conducted using quartz fiber filters (PALL Life Sciences, New York, NY, USA, TISSUQUARTZ-2500QAT-UP, 8 × 10 inches) at a flow rate of 1.13 m^3^ min^−1^, with separate collections conducted for daytime and nighttime periods. Each filter was treated in a muffle furnace (HF-Kejing Co., Hefei, China), at 780 °C for 5 h to remove organic contaminants. The weight of the filter prior to sampling was determined by taking the average of two times on a balance with a precision of 0.0001 g, then wrapped in aluminum foil that had been heated at 450 °C for 6 h and stored. After sampling, the filter was weighed using the same method, and the PM_2.5_ concentration was calculated.

### 2.2. OP Measurement

The OP of the PM_2.5_ samples was quantified by the depletion rate of DTT. Briefly, four punches (8 mm in diameter) from each filter were extracted with 4.5 mL of Milli-Q water by sonication for 30 min using an ultrasonic cleaner, and the extract was filtered into an amber glass vial. Then, 1.75 mL of the PM_2.5_ extract and 4.5 mL of phosphate buffer (0.1 mol/L, pH = 7.4) were added to a 10 mL brown centrifuge tube. The mixture was incubated in the dark at 37 °C in a water bath for 5 min. Subsequently, 0.7 mL of DTT solution (1 mmol/L) was added. At 0, 5, 10, 20, 30, 40, 50, and 60 min, 0.5 mL of the reaction mixture was withdrawn and added to a 5 mL brown centrifuge tube containing 0.5 mL of TCA solution (10% *w*/*v*). Immediately thereafter, 50 μL of DTNB solution (10 mmol/L) was added as a chromogenic reagent, followed by 2 mL of Tris-HCl buffer solution (containing 20 mmol of EDTA). The mixture was shaken well and allowed to react in the dark for 5 min. Then, 250 μL of the resulting solution was transferred into each well of a 96-well plate, and the absorbance at 412 nm was measured using a microplate reader. The absorbance values were used to calculate the DTT consumption rate for each sample.

Before the test begins, the standard solutions with concentration gradients of 0, 1, 5, 10, 25, 50, 75, and 100 μmol/L were prepared to establish the standard curve (R^2^ > 0.99) for calculating the DTT consumption rate of the PM_2.5_ samples. Samples were measured in groups of seven, with an additional ultrapure water sample included as a blank control to ensure the absorbance originated from substances in the extract. Both samples and blank controls were analyzed in triplicate.

The DTT consumption rate was normalized by dividing the DTT consumption rate by the total mass of PM_2.5_ and the collecting volume involved in the reaction, denoted as DTT_m_ (unit: pmol min^−1^ μg^−1^) and DTT_v_ (unit: nmol min^−1^ m^−3^). The DTT_m_ value indicates the intrinsic toxicity induced by PM_2.5_ and facilitates routine toxicological assessments. While the DTT_v_ value indicates the particle inhalation exposure and population exposure risk.

### 2.3. Water-Soluble Metals Analysis

For water-soluble metals analysis, one punch (47 mm diameter) from each filter was shredded into strips and placed in a brown bottle. Ultrasonic extraction was performed with ultrapure water for 60 min, divided into three sessions. The extract was then filtered using a 0.45 μm polytetrafluoroethylene (PTFE) membrane filter (Hunan BKMAN Holding Co., Ltd., Hunan, China). HNO_3_ was added to acidify the sample before measurement. The filtered extracts were analyzed for metal content using an inductively coupled plasma optical emission spectrometer (ICP-OES, Agilent 5100, Agilent Technologies, Santa Clara, CA, USA). The measured water-soluble metals included aluminum (Al), barium (Ba), calcium (Ca), copper (Cu), iron (Fe), potassium (K), magnesium (Mg), manganese (Mn), strontium (Sr), and zinc (Zn).

Prior to the analysis of environmental samples, a mixed multi-element standard solution was serially diluted with ultrapure water to obtain a series of calibration standards (0, 1, 2, 3, 4, and 5 mg L^−1^). The emission intensity of each element was measured under the optimized instrumental conditions, and calibration curves were constructed by plotting emission intensity against the corresponding metal concentration (R^2^ > 0.99). Metal concentrations in the samples were quantified using the resulting calibration curves. To ensure analytical accuracy and precision, both environmental samples and procedural blank controls were analyzed in triplicate.

### 2.4. Machine Learning Methods

Machine learning is more effective than traditional methods in modeling the complex nonlinear relationships and interactions among explanatory variables, and it can handle large datasets and noise with high predictive accuracy. This flexibility and automation make machine learning a versatile tool for analyzing variable relationships across different fields. Extreme Gradient Boosting (XGBoost), proposed by Chen and Guestrin from the University of Washington, is an improved version of the traditional Gradient Boosted Decision Trees (GBDT) algorithm [33]. Based on the gradient boosting framework, XGBoost iteratively weights the residuals of the training data, with each newly constructed decision tree aimed at correcting the prediction errors of the previous round, thereby continuously enhancing overall model performance [34,35]. In addition, the algorithm excels in parallel computing efficiency, handling of missing values, overfitting control, and generalization capability, making it widely applied across various research fields [35]. To more accurately quantify the contributions of target factors, the SHAP algorithm is employed. Based on Shapley values from game theory, this method evaluates the importance of each feature for individual predictions, offering a systematic approach to assess how each variable influences the model’s output [36,37].

To investigate the nonlinear relationships between PM_2.5_ oxidative potential (OP) and atmospheric pollutants, an interpretable machine-learning framework based on Extreme Gradient Boosting and SHAP (XGBoost–SHAP) was employed. OP was selected as the target variable. Predictor variables included twelve measured chemical components and three gaseous pollutants (SO_2_, NO_2_, and O_3_). Meteorological parameters were not included in the model, as the primary objective was to identify the chemical drivers directly associated with OP variability.

The dataset was randomly divided into training (70%), validation (15%), and testing (15%) subsets without replacement using a fixed random seed to ensure reproducibility. All datasets were converted into the DMatrix format required by the XGBoost package. Hyperparameters, including maximum tree depth (max_depth), learning rate (eta), and number of boosting rounds (nrounds), were optimized based on the performance of the training and validation datasets. A watchlist containing both training and validation datasets was used during model training to monitor model convergence and reduce the risk of overfitting.

Model performance was evaluated using the coefficient of determination (R^2^) and root mean square error (RMSE). The final model achieved an R^2^ of 0.916 and an RMSE of 0.361 for the training dataset, while the testing dataset yielded an R^2^ of 0.750 and an RMSE of 0.576, indicating good predictive performance and satisfactory generalization ability. SHAP (Shapley Additive exPlanations) analysis was subsequently applied to quantify feature importance and interpret the contribution of individual predictors to OP variability.

### 2.5. Positive Matrix Factor (PMF)

EPA PMF 5.0 is widely used to apportion source-specific contributions to atmospheric aerosols, with comprehensive methodological details provided in the EPA PMF 5.0 Rationale and User’s Guide [38]. Prior to running the model, input data were carefully screened to eliminate irrelevant components and outliers, thereby minimizing their potential interference with the results. Components with a signal-to-noise ratio (S/N) less than 0.5 were classified as ‘Bad’ and excluded from the analysis. Components with S/N between 0.5 and 1 were considered ‘Weak’ introducing greater uncertainty into the analysis. Components with S/N greater than 1 were classified as ‘Strong’. The uncertainty value is calculated using Equation (1) when the concentration of a given chemical species exceeds the method detection limit (MDL); otherwise, it is calculated using Equation (2).

(1) $$\mathrm{Unc}=\sqrt{{(Error\;Fraction\times C)}^{2}+{(0.5\times \mathrm{MDL})}^{2}}$$

(2) $$\mathrm{Unc}=\frac{5}{6}\times \mathrm{MDL}$$

## 3. Results and Discussion

### 3.1. Air Pollution and OP of PM_2.5_ During the Study Period

**Figure 1:**
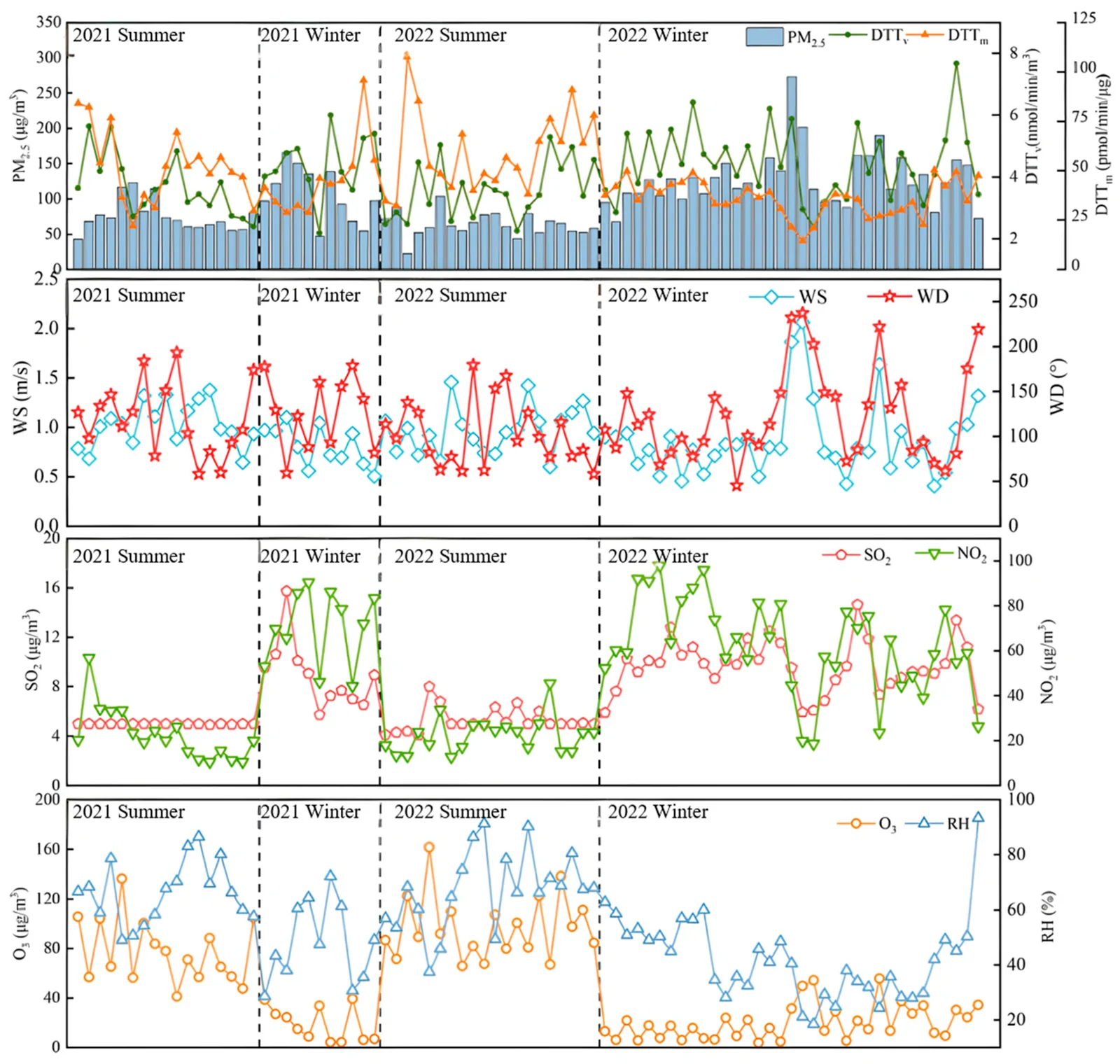
Time series of atmospheric gaseous pollutants, meteorological variables, PM_2.5_ mass concentration, and OP.

Figure 1 shows the temporal variations in meteorological parameters (wind speed (WS), wind direction (WD), and relative humidity (RH)), gaseous pollutants (SO_2_, NO_2_, and O_3_), PM_2.5_ mass concentration, and oxidative potential (OP) during the study period in Xi’an.

As shown in Figure 1, PM_2.5_ mass concentration and OP exhibited pronounced temporal and seasonal variations throughout the study period. PM_2.5_ concentrations were substantially higher in winter than in summer, particularly during the heating seasons of 2021 and 2022, when frequent pollution episodes occurred.

Unlike PM_2.5_ mass concentration, OP exhibited a distinctly different temporal pattern. DTT_v_ was markedly higher in winter, indicating an increased oxidative burden per unit air volume during winter. In contrast, DTT_m_ exhibited considerable temporal variability and did not consistently track changes in PM_2.5_ mass concentration. Several periods with only moderate PM_2.5_ concentrations exhibited relatively high DTT_m_ values, suggesting that intrinsic oxidative toxicity varied over time and could not be explained solely by PM_2.5_ mass concentration.

Meteorological conditions and gaseous pollutants also exhibited clear seasonal patterns (Figure 1). Winter was characterized by lower wind speeds and higher SO_2_ and NO_2_ concentrations, consistent with enhanced combustion emissions, whereas O_3_ concentrations were higher in summer. Periods of elevated DTT_v_ frequently coincided with higher SO_2_ and NO_2_ concentrations, suggesting a potential contribution of combustion-related sources to PM_2.5_ oxidative potential.

Overall, Figure 1 shows that seasonal changes in PM_2.5_ pollution were accompanied by disproportionate changes in oxidative potential, highlighting that OP provides additional toxicologically relevant information beyond PM_2.5_ mass concentration alone.

**Figure 2:**
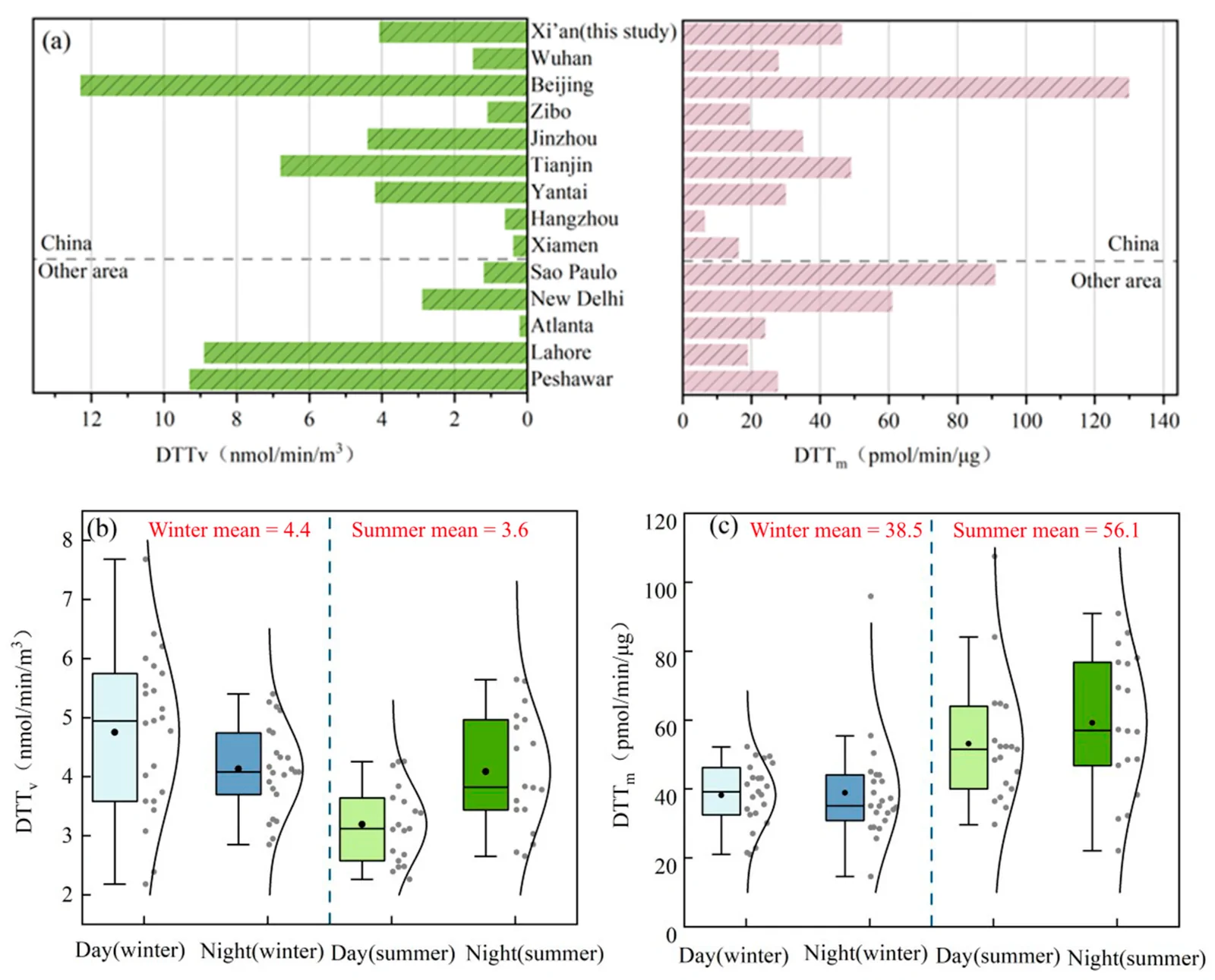
(**a**) The OP of PM_2.5_ measured in this study is compared with those reported in previous studies conducted in other cities worldwide. (**b**,**c**) Box plots of the seasonal and diurnal DTT_V_ and DTT_m_ values with the annual average value.

As shown in Figure 2a, the average DTT_v_ and DTT_m_ values of PM_2.5_ were 4.1 ± 1.1 nmol min^−1^ m^−3^ and 46.4 ± 18.0 pmol min^−1^ μg^−1^, respectively. Compared with previous studies worldwide, the DTT_v_ observed in Xi’an was higher than those reported for Atlanta [39], Delhi [40], and Sao Paulo [21] but lower than those reported for Lahore and Peshawar (Figure 2). Interestingly, although Lahore and Peshawar exhibited higher DTT_v_ values, their DTT_m_ values were approximately half of those measured in Xi’an [41]. This discrepancy suggests that population-level oxidative exposure (DTT_v_) and intrinsic particle toxicity (DTT_m_) do not necessarily scale proportionally and are strongly influenced by regional differences in PM composition and sources.

Compared with other studies conducted in China, the DTT_v_ and DTT_m_ values observed in the present study were comparable to those reported for Jinzhou, Tianjin and Yantai [30], slightly higher than those measured in Nanjing, Xi’an, Beijing, Guangzhou, Hangzhou and Shanghai [42], and substantially higher than those in Xiamen [31]. These spatial differences in OP highlight the heterogeneity of PM_2.5_ oxidative toxicity across China, which cannot be explained by particle mass concentration alone. In northern cities such as Xi’an, Beijing, and Tianjin, wintertime heating and enhanced combustion activities may substantially alter PM chemical composition, leading to elevated oxidative potential compared with southern cities. In addition, differences in PM extraction procedures may also contribute to inter-study variability in OP measurements [43].

Seasonal and diurnal variations in OP are illustrated in Figure 2b,c. Considering all samples collected within each season, the mean DTT_v_ was 4.4 ± 1.1 nmol min^−1^ m^−1^ in winter and 3.6 ± 0.92 nmol min^−1^ m^−3^ in summer, indicating a stronger oxidative stress–inducing capacity of PM_2.5_ during the winter. Similar seasonal patterns have been reported in Kuwait [44] and Chamonix [45], and are generally attributed to increased combustion emissions and enhanced partitioning of redox-active semi-volatile organic compounds (SVOCs) into the particle phase under lower temperatures [35]. In contrast, lower DTT_v_ values in summer may result from reduced local emissions, dominance of aged aerosols, and volatilization of SVOCs under high temperatures [44,45,46].

Distinct diurnal variations in OP were also observed. In winter, daytime DTT_v_ values consistently exceeded nighttime values, which may be associated with increased PM_2.5_ emissions from traffic and human activities during the day [30]. However, no significant diurnal difference was observed for DTT_m_ in winter, suggesting that the intrinsic oxidative toxicity of particles remained relatively stable. In summer, both DTT_v_ and DTT_m_ were higher at night, potentially due to daytime volatilization of redox-active organic compounds under high temperatures, leading to reduced particle-phase oxidative activity [44].

Overall, these findings demonstrate pronounced seasonal and diurnal variations in the oxidative potential of PM_2.5_ in Xi’an, reflecting dynamic changes in emission sources and chemical composition. Importantly, elevated OP values, particularly DTT_v_, imply an enhanced capacity of ambient PM_2.5_ to induce oxidative stress and related toxic effects, underscoring the toxicological relevance of OP beyond traditional mass-based metrics.

The elevated wintertime DTT_v_ observed in Xi’an likely reflects not only higher PM_2.5_ concentrations but also seasonal shifts in particle composition. Winter heating, biomass burning, and stagnant meteorological conditions promote the accumulation and atmospheric aging of redox-active species, including transition metals and combustion-derived organic compounds. Consequently, the greater oxidative burden observed in winter is likely attributable to both increased PM_2.5_ loading and enrichment of highly redox-active chemical constituents [47,48,49].

### 3.2. Seasonal Variations in Water-Soluble Metals

To better understand the compositional factors governing PM_2.5_ oxidative potential, the seasonal characteristics of water-soluble metals were first examined. During the study period, the total concentration of water-soluble metals in PM_2.5_ collected in Xi’an was 2830.3 ± 1691.3 ng/m^3^, accounting for approximately 2.9% of the total PM_2.5_ mass. This proportion is comparable to that reported by Li et al. [31], who found that nine water-soluble metals accounted for 3.4% of PM_2.5_ mass in Xiamen. This consistency suggests that water-soluble metals contribute a similar fraction of PM_2.5_ across urban environments in China. Although their mass fraction is relatively low, water-soluble metals are considered toxicologically important because of their high bioavailability and their ability to participate in redox cycling reactions that promote ROS generation after particle deposition in the respiratory tract [50,51,52].

Table 1 summarizes the seasonal variations in water-soluble metal concentrations in PM_2.5_ collected in Xi’an. The average concentrations decreased in the following order: Ca > K > Mg > Al > Fe > Zn > Cu > Ba > Mn > Sr. Among these elements, Ca was the most abundant, with an average concentration of 2016.9 ± 1370 ng/m^3^, accounting for approximately 70% of the total water-soluble metal content. Its concentration exceeded that of any other metal by more than one order of magnitude. The increasing concentrations of Ca, together with Mg and Sr, from summer to winter suggest a strong influence of mineral dust sources. These particles may act as carriers of toxic trace metals, facilitating their atmospheric transport and subsequent interaction with biological systems [53].

Pronounced seasonal differences were observed for several metals. The concentrations of Ca, K, Mg, and Sr were more than twofold higher in winter than in summer. The elevated wintertime concentration of K indicates a substantial contribution from biomass burning [41], which emits abundant water-soluble species that are closely associated with enhanced PM_2.5_ oxidative potential. Al, Mg, Ca, Fe, Mn, Sr, and Ba are widely recognized as tracers of soil and resuspended dust emissions. Their higher wintertime concentrations may be attributed to surface drying, enhanced dust resuspension, and stagnant meteorological conditions that promote pollutant accumulation [54,55].

In addition, Ba, Fe, Mn, and Zn are also recognized tracers of metal manufacturing activities, particularly the steel industry [54]. The higher wintertime Fe concentration likely reflects the combined influence of dust resuspension and industrial emissions [54,55]. By contrast, Ba and Mn exhibited relatively limited seasonal variation, with slightly higher concentrations in winter. Cu and Zn, recognized as tracers of non-exhaust traffic-related emissions, including brake wear, tire wear, and asphalt abrasion [55], showed slightly higher concentrations in summer than in winter. These metals are of particular toxicological interest because of their high solubility and strong redox activity, which facilitate ROS generation.

**Figure 3:**
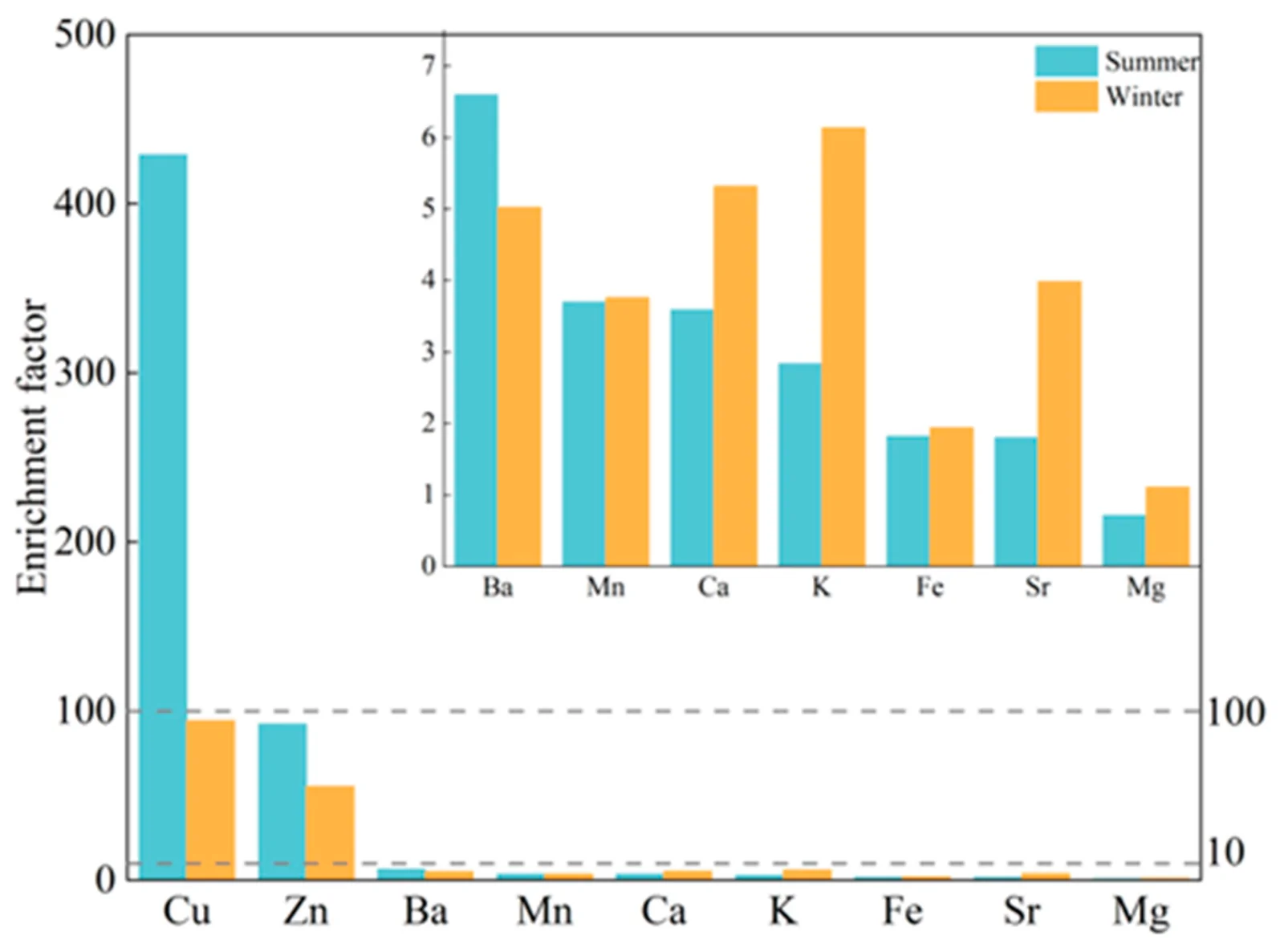
Enrichment factors of metal in atmospheric PM_2.5_ in Xi’an during winter and summer.

Figure 3 presents the enrichment factors (EFs) of water-soluble metals in PM_2.5_ collected in Xi’an. An EF ≤ 1 indicates no significant enrichment, suggesting that the metal is primarily derived from crustal or soil sources. An EF between 1 and 10 indicates slight enrichment, implying contributions from both natural and anthropogenic sources. An EF between 10 and 100 indicates moderate enrichment, suggesting that anthropogenic activities are the dominant source of the metal. For clarity, the EF classification thresholds are shown as dashed horizontal lines in Figure 3. Mg exhibited EF values close to unity, indicating minimal enrichment and predominantly crustal or soil-derived sources. Ba, Mn, Ca, K, Fe, and Sr exhibited EF values between 1 and 10, suggesting slight enrichment and mixed contributions from natural and anthropogenic sources, including biomass burning, traffic-related emissions, and brake wear. Among these elements, Ca, K, Fe, and Sr exhibited higher EF values in winter than in summer, indicating enhanced atmospheric accumulation during winter.

Notably, the EF of K increased markedly in winter, further supporting an enhanced contribution from biomass burning. Biomass burning emits abundant water-soluble potassium and other redox-active species that can substantially increase PM_2.5_ oxidative potential. Zn exhibited EF values ranging from 10 to 100 in winter and exceeding 100 in summer, whereas Cu exhibited EF values greater than 100 in both seasons. The pronounced enrichment of Cu and Zn, particularly in summer, indicates dominant anthropogenic sources, primarily associated with non-exhaust traffic-related emissions. Given their high solubility and strong redox activity, the pronounced enrichment of Cu and Zn suggests that traffic-related emissions may contribute disproportionately to PM_2.5_ oxidative toxicity, particularly in summer. These findings provide important compositional evidence for the subsequent analysis of the major drivers of oxidative potential.

### 3.3. Relationship Between Water-Soluble Metals and OP

**Figure 4:**
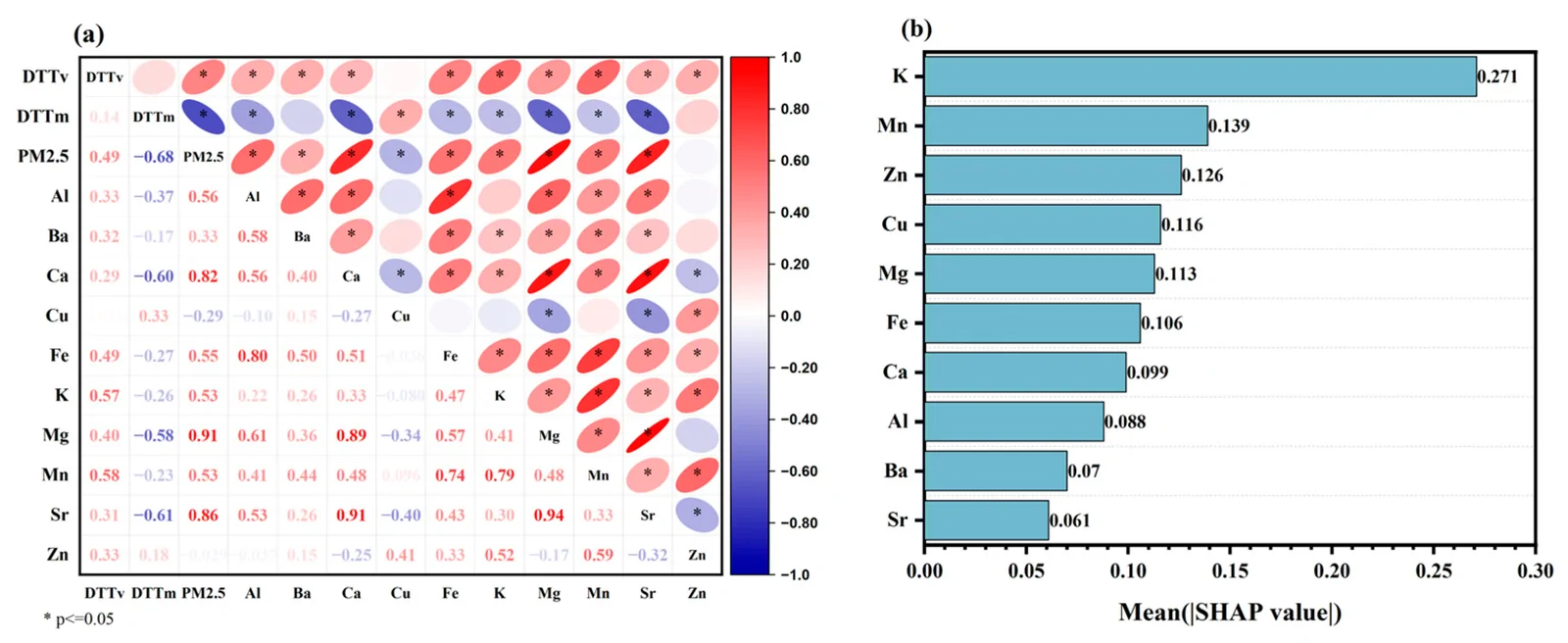
(**a**) Pearson correlation coefficients between DTT_v_, DTT_m_, and water-soluble metals. (**b**) The mean SHAP values calculated from the XGBoost-SHAP model.

Based on the observed seasonal variations in metal composition, correlation analysis and machine-learning modeling were performed to identify the major chemical drivers of PM_2.5_ oxidative potential. Figure 4a shows the relationships among PM_2.5_ mass concentration, water-soluble metals, and OP metrics. DTT_m_ was negatively correlated with PM_2.5_ concentration. A nonlinear decreasing trend was further identified by curve fitting (Figure S1), suggesting that components contributing substantially to PM mass do not necessarily increase the intrinsic ROS-generating capacity of particles [23]. By contrast, DTT_v_ was positively correlated with PM_2.5_ concentration (R^2^ = 0.49), indicating a greater oxidative burden per unit air volume at higher PM_2.5_ levels. This metric is widely recognized as toxicologically relevant and has been associated with adverse health outcomes [18].

Both DTT_v_ and DTT_m_ were positively correlated with several water-soluble metals, particularly K, Fe, Mn, and Mg. These consistent associations support the role of redox-active metals in promoting ROS generation within PM, thereby enhancing oxidative potential. Transition metals such as Fe, Mn, Cu, V, and Ni can generate H_2_O_2_ and hydroxyl radicals (•OH) through Fenton-like reactions [56,57,58]. Although Cu was not significantly correlated with DTT_v_ across all samples, seasonal analysis (Table S1) revealed a positive correlation in winter. This finding suggests a season-dependent contribution of non-exhaust traffic-related and combustion emissions to PM_2.5_ oxidative potential.

Notably, the correlation between K and DTT_v_ was stronger in winter (R^2^ = 0.54) than in summer (R^2^ = 0.42). Because K is a well-established tracer of biomass burning, this result further supports biomass combustion as a major contributor to the elevated PM_2.5_ oxidative potential observed in winter. By contrast, crustal elements such as Al, Mg, Ca, and Sr showed no significant correlation with DTT_v_ despite their relatively high concentrations and exhibited weak or negative correlations with DTT_m_. This pattern may reflect collinearity between crustal elements and PM_2.5_ concentration, which was itself negatively associated with DTT_m_ [31].

Because the relationships between OP and PM chemical composition are complex and potentially nonlinear, correlation analysis alone may not fully capture the contribution of individual components. Therefore, an XGBoost–SHAP model was employed to further quantify the relative importance of water-soluble metals. As shown in Figure 4b, K and Mn exhibited the highest SHAP values, identifying them as the most influential contributors to OP. Variations in their concentrations exerted a disproportionate influence on particle oxidative potential. K primarily originates from biomass burning, further supporting the substantial contribution of combustion sources to PM oxidative potential. Mn also exhibited a pronounced influence on OP. This effect may be attributed to its synergistic interactions with organic compounds, including humic-like substances and quinones, during DTT consumption and ROS generation [11]. In addition, Cu can promote H_2_O_2_ production in the presence of quinones [59], whereas Fe enhances Fenton reactions through redox cycling, generating highly reactive •OH radicals. Together, these processes substantially increase particle oxidative potential and reinforce the toxicological relevance of transition metals [14,60,61,62,63].

Interestingly, Mn exhibited greater SHAP importance than several metals traditionally associated with DTT activity, including Fe. This finding suggests that the contribution of metals to oxidative potential depends not only on their concentration but also on their solubility, redox cycling efficiency, and interactions with coexisting particle constituents. Previous studies have demonstrated that Mn promotes ROS generation through catalytic redox cycling [64]. Emerging evidence further suggests that interactions between transition metals and combustion-derived organic components, including quinones and humic-like substances (HULIS), can further enhance particle oxidative potential [65]. Consequently, even modest variations in Mn concentration may produce disproportionately large changes in oxidative potential.

### 3.4. Source Contributions to Oxidative Potential

**Figure 5:**
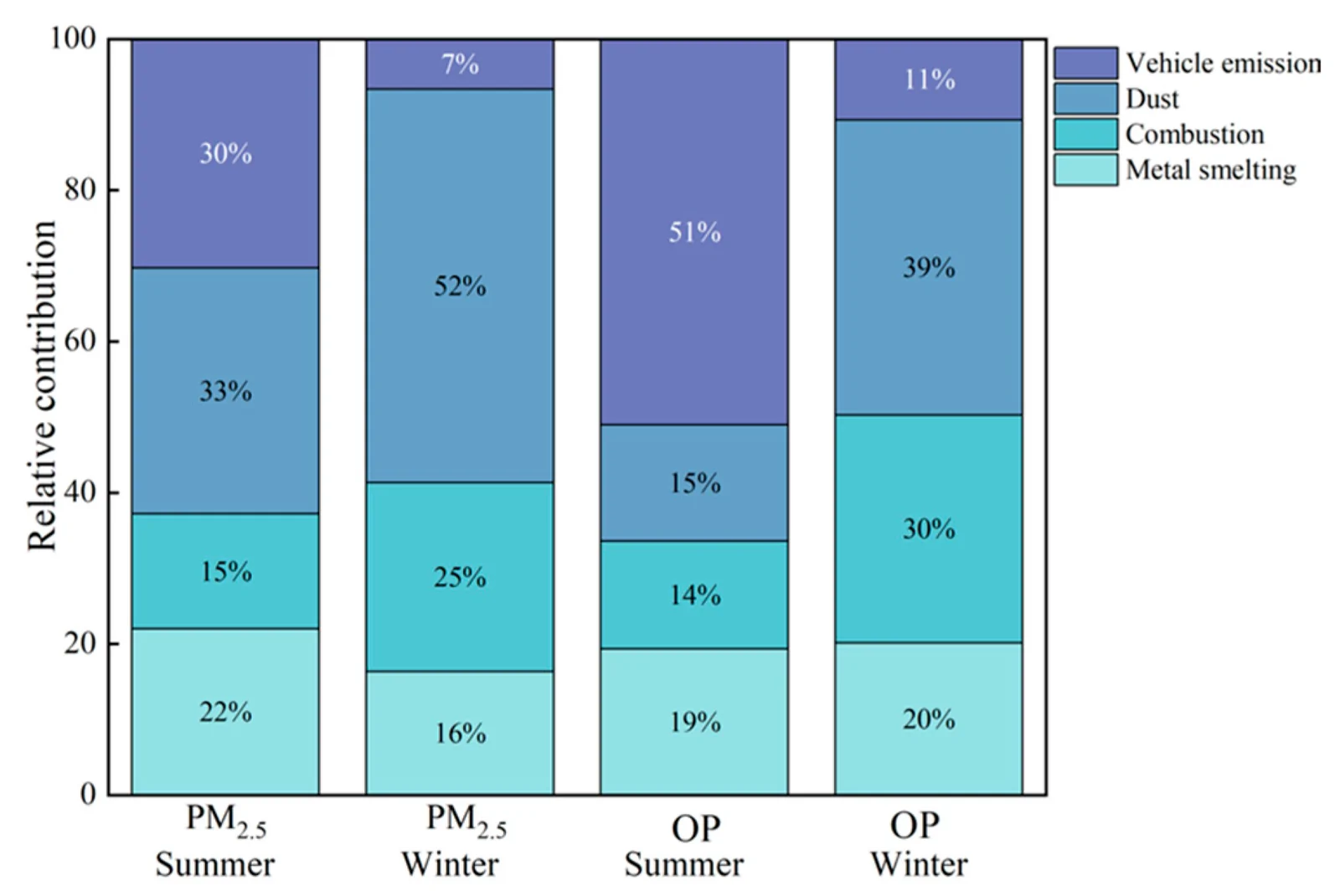
Seasonal variations in PM_2.5_ and OP sources.

After identifying the major oxidative components, source apportionment analysis was conducted to determine the emission sources driving PM_2.5_ oxidative potential. The PMF model resolved four major sources contributing to PM_2.5_ at the sampling sites. The corresponding PMF source profiles are presented in Figure S1. Figure 5 shows the seasonal contributions of different sources to PM_2.5_ mass and oxidative potential (OP). Dust sources were the largest contributors to PM_2.5_ mass, accounting for 52% in winter and 32.5% in summer. These sources primarily comprised construction dust, road dust, and resuspended particles generated by transportation activities. During winter pollution episodes, the contribution of fugitive dust increased markedly. This finding reflects the strong influence of stagnant meteorological conditions and surface disturbance on PM_2.5_ mass loading rather than on intrinsic oxidative toxicity.

Combustion sources accounted for 25% of PM_2.5_ mass in winter and 15.2% in summer, making them the second-largest source during winter. The greater wintertime contribution is likely attributable to increased heating demand and intensified combustion activities, which substantially increase PM_2.5_ concentrations. Metal smelting sources accounted for 22% of PM_2.5_ mass in summer and 16.3% in winter, representing emissions from industrial combustion and manufacturing activities. Despite their lower wintertime contribution, metal smelting remained an important source of PM_2.5_ throughout the year. Traffic-related emissions contributed only 6.6% of PM_2.5_ mass in winter but increased to 30.3% in summer, becoming the second-largest source. The greater contributions of combustion and dust sources during winter reduced the relative contribution of traffic-related emissions.

Based on the PMF-resolved source profiles and their contributions to measured PM_2.5_, a multiple linear regression (MLR) model was applied to quantify source-specific contributions to OP. The MLR results are summarized in Table S2. Although dust sources exhibited the lowest intrinsic oxidative activity, they accounted for 30% of the estimated total DTT_v_ activity because of their dominant contribution to PM_2.5_ mass. This apparent discrepancy reflects the dominant contribution of dust to PM_2.5_ mass, particularly in winter, when it accounted for more than half of the total particulate load (52%).

Combustion sources contributed more substantially to DTT_v_ in winter. Located in northwestern China, Xi’an is strongly influenced by open biomass burning and residential biomass combustion for cooking and heating during winter. These activities represent important sources of combustion-derived aerosols. By contrast, traffic-related emissions were the dominant contributors to OP during summer, accounting for 51% of the total DTT_v_ activity, compared with 11% in winter. This pronounced seasonal difference suggests that traffic-related emissions exhibit higher intrinsic oxidative activity during summer. Raparthi et al. [66] reported that carbonaceous aerosols from traffic-related emissions are important drivers of ROS generation because quinones and polycyclic aromatic hydrocarbons (PAHs) promote the formation of superoxide (O_2_•^−^) and hydroxyl (•OH) radicals through redox cycling and Fenton-like reactions. Similarly, Li et al. [67] found that elevated levels of black carbon (BC) and heavy metals from traffic-related emissions were major contributors to PM_2.5_ OP in Beijing.

The contribution of metal smelting sources to DTT_v_ showed little seasonal variation, indicating a relatively stable contribution to oxidative potential throughout the year. This result suggests that although the mass contribution of metal-related emissions varies seasonally, their overall oxidative activity remains relatively consistent, underscoring the persistent toxicological relevance of metal-related sources to PM_2.5_ oxidative potential.

The discrepancy between source contributions to PM_2.5_ mass and oxidative potential has important implications for air-quality management. Conventional pollution-control strategies primarily target reductions in PM_2.5_ mass concentration. However, the present results indicate that sources contributing relatively little to particle mass may exert disproportionately large effects on oxidative potential. Therefore, mitigation strategies prioritizing traffic-related emissions and combustion sources may provide greater public health benefits than approaches focusing solely on reducing PM_2.5_ mass.

## 4. Conclusions

Several limitations of this study should be acknowledged. First, oxidative potential was evaluated solely using the DTT assay, which primarily reflects the activity of specific redox-active components and may not capture all oxidative mechanisms. Second, this study focused only on water-soluble metals, whereas organic species such as quinones and PAHs may also contribute substantially to oxidative potential. Third, samples were collected at a single urban site, which may limit the spatial representativeness of the results. Future studies integrating multiple oxidative-potential assays, more comprehensive chemical characterization, and multi-site observations would provide a more complete understanding of PM_2.5_ oxidative potential and its associated health risks.

This study evaluated the oxidative potential (OP) of ambient PM_2.5_ in Xi’an using the dithiothreitol (DTT) assay and investigated its chemical drivers and emission sources by integrating correlation analysis, XGBoost–SHAP modeling, PMF source apportionment, and multiple linear regression. Pronounced seasonal differences in OP were observed, with significantly higher DTT_v_ values in winter, indicating a greater oxidative burden associated with ambient PM_2.5_ exposure during the heating period. The results demonstrate that PM_2.5_ oxidative potential is governed more by chemical composition than by particle mass concentration. Potassium, manganese, and iron were identified as the major contributors to OP, highlighting the important roles of biomass burning and redox-active transition metals in particle-associated ROS generation.

Source-oriented analysis further revealed that traffic-related and combustion emissions contributed disproportionately to oxidative potential relative to their contributions to PM_2.5_ mass, whereas dust sources, despite dominating particle mass, exhibited comparatively low intrinsic oxidative activity. These findings emphasize that reducing PM_2.5_ mass alone may not effectively mitigate the health risks associated with particulate pollution. The integrated PMF–SHAP framework developed in this study provides a robust approach for identifying the chemical and source-specific drivers of PM_2.5_ oxidative potential and offers scientific support for toxicity-oriented air-quality management and targeted emission-control strategies. These findings highlight the importance of incorporating toxicity-based metrics into future air-quality assessment and pollution-control strategies, complementing conventional PM_2.5_ mass-based standards.

## Figures and Tables

**Table 1:** The concentration of water-soluble metals in Xi’an during winter and summer.

Species (ng/m^3^)	Summer		Winter	
	Mean	Standard	Mean	Standard
Al	47.8	30.0	72.9	40.7
Ba	14.0	12.1	16.3	6.9
Ca	1202.7	873.1	2720.3	1375.3
Cu	50.5	36.5	17.0	18.6
Fe	42.5	30.9	69.1	35.7
K	189.6	81.9	625.6	425.6
Mg	74.6	34.4	175.9	85.4
Mn	10.6	5.4	16.5	7.3
Sr	2.9	1.8	9.8	5.3
Zn	39.7	24.8	36.3	48.0

## Data Availability

The original contributions presented in this study are included in the article/Supplementary Material. Further inquiries can be directed to the corresponding author.

## Supplementary Materials

The following supporting information can be downloaded at https://www.mdpi.com/article/10.3390/toxics14080646/s1. Figure S1: Compositional profiles (species percentage and concentration) corresponding to the factors resolved by the PMF model. Table S1: The correlation between OP of atmospheric PM_2.5_ and water-soluble metals. Table S2: The MLR analysis results between DTT_v_ and PM_2.5_ sources.
