# Supplementary material for: Source-Specific Oxidative Potential of PM2.5 in Xi’an: Roles of Water-Soluble Metals Revealed by DTT Assay and Interpretable Machine Learning
Source: Toxics. 2026 Jul 23;14(8):646. doi: 10.3390/toxics14080646 (PMC13517877; doi:10.3390/toxics14080646)
Supplement: Supplementary file 1 [file toxics-14-00646-s001.zip › toxics-4347920-supplementary.pdf]

Supplementary Materials

Source-Specific Oxidative Potential of $PM_{2.5}$ in Xi'an: Roles of Water-Soluble Metals Revealed by DTT Assay and Interpretable Machine Learning

Lei Chen [1,2], Na Wang [1], Qian Zhang [1], Xinghua Zhang [3], Zhihua Li [1] and Weidong Jing [1,*]

[1] School of Environmental and Municipal Engineering, Xi'an University of Architecture and Technology, Xi'an 710055, China
[2] Department of Ecology and Environment of Shaanxi Province, Xi'an 710006, China
[3] Shaanxi Key Laboratory of Earth Surface System and Environmental Carrying Capacity, College of Urban and Environmental Sciences, Northwest University, Xi'an 710127, China

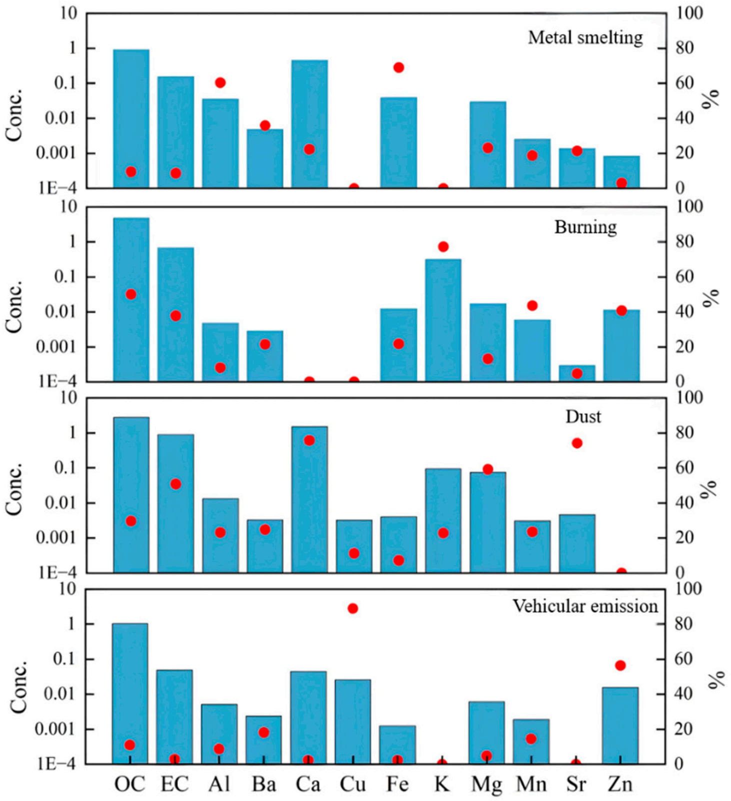

Figure S1. Compositional profiles (species percentage and concentration) corresponding to the factors resolved by the PMF model. The blue bars and red solid circles represent the concentrations and percentages of different species, respectively.

Table S1. The correlation between OP of atmospheric $PM_{2.5}$ and water-soluble metals

	Winter		Summer	
	DTT_v	DTT_m	DTT_v	DTT_m
Al	0.24	-0.34*	0.25	-0.20
Ba	0.38**	-0.13	0.28	-0.13
Ca	0.19	-0.52**	-0.035	-0.5**
Cu	0.37*	0.14	0.24	0.09
Fe	0.43**	-0.27	0.4**	0.029
K	0.54**	0.054	0.42**	-0.094
Mg	0.28	-0.58**	0.18	-0.34*
Mn	0.56*	-0.078	0.42**	-0.011
Sr	0.13	-0.61**	0.045	-0.45**
Zn	0.36*	0.14	0.46**	0.31

Table S2. The MLR analysis results between DTT_v and $PM_{2.5}$ sources

Model	Unstandardized coefficients	Standardized coefficients	t-STAT	p-Value
	B + Standard error	Beta		
1 n=83,r^2=0.639				
Metal smelting	0.161+0.13	0.802	12.150	0.000
2 n=83,r^2=0.796				
Metal smelting	0.102+0.12	0.507	8.220	0.000
Combustion	0.072+0.009	0.495	8.011	0.000
3 n=83,r^2=0.844				
Metal smelting	0.084+0.011	0.418	7.367	0.000
Combustion	0.059+0.008	0.410	7.253	0.000
Vehicle emission	0.065+0.013	0.270	5.101	0.000
4 n=83,r^2=0.908				
Metal smelting	0.045+0.010	0.224	4.429	0.000
Combustion	0.043+0.007	0.297	6.488	0.000
Vehicle emission	0.077+0.10	0.321	7.782	0.000
Dust	0.025+0.003	0.355	7.546	0.000
